# Urban weather data and building models for the inclusion of the urban heat island effect in building performance simulation

**DOI:** 10.1016/j.dib.2017.08.035

**Published:** 2017-08-31

**Authors:** M. Palme, L. Inostroza, G. Villacreses, A. Lobato, C. Carrasco

**Affiliations:** a Universidad Católica del Norte, Antofagasta, Chile; b Instituto Nacional de Eficiencia Energética y Energías Renovables, Quito, Ecuador; c Institute of Geography, Ruhr Universität Bochum, Germany; d Universidad Autónoma de Chile, Temuco, Chile; e Universidad de Valparaíso, Chile

**Keywords:** Building performance simulation, Urban heat island, Geographical Information Systems, Urban Weather Generator, Principal Component Analysis

## Abstract

This data article presents files supporting calculation for urban heat island (UHI) inclusion in building performance simulation (BPS). Methodology is used in the research article “From urban climate to energy consumption. Enhancing building performance simulation by including the urban heat island effect” (Palme et al., 2017) [1]. In this research, a Geographical Information System (GIS) study is done in order to statistically represent the most important urban scenarios of four South-American cities (Guayaquil, Lima, Antofagasta and Valparaíso). Then, a Principal Component Analysis (PCA) is done to obtain reference Urban Tissues Categories (UTC) to be used in urban weather simulation. The urban weather files are generated by using the Urban Weather Generator (UWG) software (version 4.1 beta). Finally, BPS is run out with the Transient System Simulation (TRNSYS) software (version 17). In this data paper, four sets of data are presented: 1) PCA data (excel) to explain how to group different urban samples in representative UTC; 2) UWG data (text) to reproduce the Urban Weather Generation for the UTC used in the four cities (4 UTC in Lima, Guayaquil, Antofagasta and 5 UTC in Valparaíso); 3) weather data (text) with the resulting rural and urban weather; 4) BPS models (text) data containing the TRNSYS models (four building models).

**Specifications Table**

**Table:** 

Subject area	*Building Simulation, Spatial Analysis*
More specific subject area	*Principal Component Analysis, Urban Weather Generation; Trnsys simulation.*
Type of data	*Weather data (xls), Building Performance Simulation Models (txt), Urban Simulation Models (txt). Figures, tables and text.*
How data was acquired	*Rural weather data were obtained by using Meteonorm software (Guayaquil, and Valparaiso) and from Energy Plus website (Lima and Antofagasta) in Energy Plus Weather (EPW) format. UWG and TRNSYS models have been developed by the authors.*
Data format	*Derived data, Analyzed data.*
Experimental factors	*Urban morphology was recovered from Archgis and Google Street View. PCA was run out with excel tool.*
Experimental features	*Urban weather data have been generated as.epw by using UWG software.*
Data source location	*Guayaquil, Ecuador; Lima, Peru; Antofagasta, Chile; Valparaíso, Chile.*
Data accessibility	*Data are available within this article.*

**Value of the data**

• Presented urban weather data enable researchers to improve building simulations for the cities of Lima, Guayaquil, Valparaiso and Antofagasta by considering the UHI effect.
• Presented PCA data enable researchers to downscale urban climate to building level for building performance simulation purposes in other locations of the world.
• Presented UWG data permit to generate similar urban scenarios in other locations of the world.
• Building Performance Simulation models are useful to conduct similar building simulations in other locations of the world.

## 1 Data

Presented data are files needed for the inclusion of urban heat island (UHI) in building performance simulation (BPS). BPS needs weather files to obtain the thermal demand of buildings; normally weather files are standard files, obtained from monitoring stations close to the location. However, urban climate is different from this standard climate, which is often a rural climate (meteorological stations are normally placed in airports or in other city's surroundings). To include UHI effect, urban climate has to be downscaled to building level by a four steps methodology [1]. In Supplementary materials, four sets of data are accessible: PCA data, UWG data, EPW data and TRNSYS models. Each of these sets is needed in one of the four steps of methodology described in the follow.

**Fig. 1:**
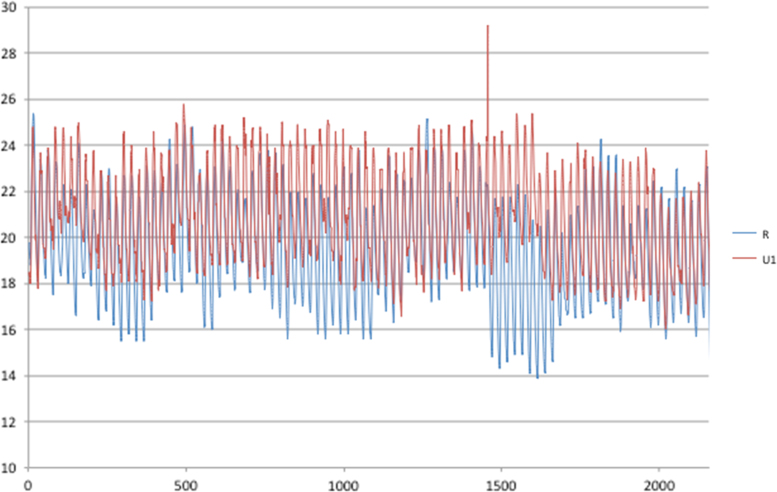
Rural and urban temperatures for Antofagasta, 90 days (1/1–31/3).

**Fig. 2:**
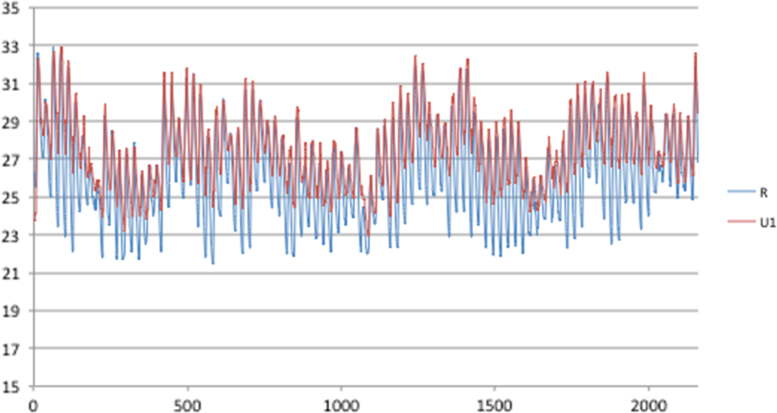
Rural and urban temperatures for Guayaquil, 90 days (1/1–31/3).

**Fig. 3:**
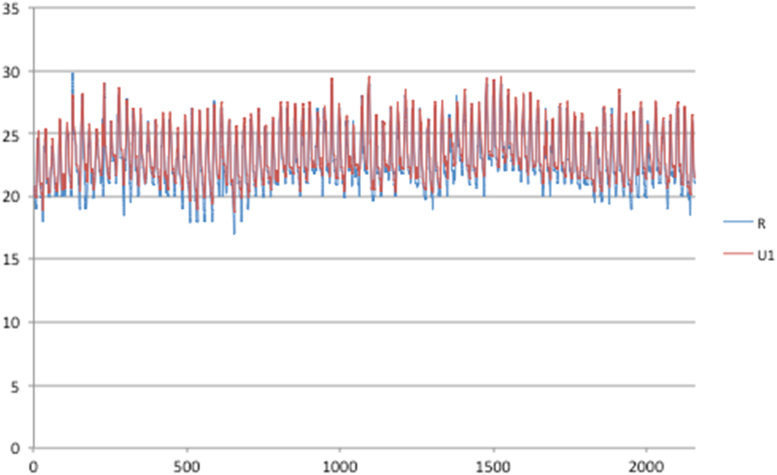
Rural and urban temperatures for Lima, 90 days (1/1–31/3).

**Fig. 4:**
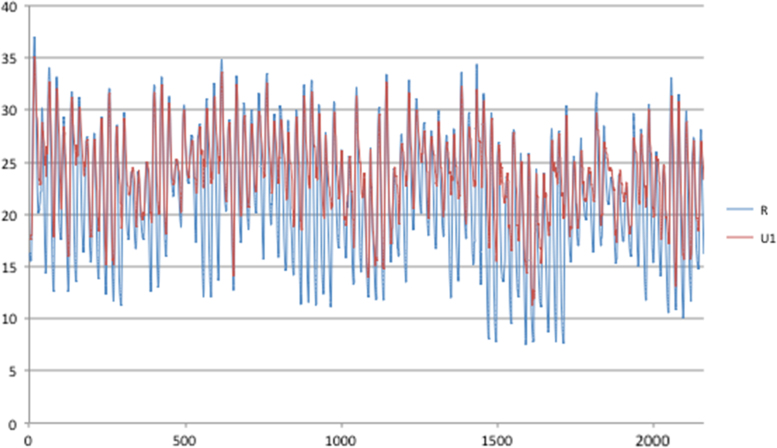
Rural and urban temperatures for Valparaiso, 90 days (1/1–31/3).

PCA: 4 excel files (one for each city), containing urban parameters (built up area, green area, façade ratio, materials of surfaces) for each sample and the following Principal Component analysis done to group the samples.
UWG: 17 Extensible Markup Language (xml) files containing the urban description of each group (4 for Lima, Antofagasta and Guayaquil and 5 for Valparaiso).
TRNSYS: 3 models for building performance simulation in.bld format and 3 simulation studio files for Trnsys.
EPW: 21 weather files containing the information needed to run building simulation (temperature, humidity, solar radiation, wind, etc.). 4 files are rural and 17 are urban (one for each group described above). Fig. 1, Fig. 2, Fig. 3, Fig. 4 show the 90 days temperature profiles for rural and urban climates in each city studied. The data can be used to visualize the urban microclimate and to include UHI in BPS.

## 2 Experimental design, materials and methods

To generate urban weather files to be used in building performance simulation, a four step methodology was developed: 1) cities are divided in zones of influence of the Sea and then a random distribution of 24 samples is done; 2) each sample was analyzed by using Archgis and Google Street View to obtain some important urban parameter (like built up area, green area and façade ratio in each sample); 3) a PCA [2], [3] analysis is done to avoid correlation between variables and to group the samples in similar urban tissues categories; 4) UWG [4], [5] is used to obtain weather files to be inserted in BPS (done by using TRNSYS [6]).

TRNSYS is a validated tool to conduct BPS; UWG is a relatively new tool useful to obtain urban weather files. However, the parameters needed by UWG have to be obtained from direct observation of urban morphology. The use of Urban Tissue Categories enables to represent a city with a relatively small number of morphologies. Each of these morphologies can be translated into a specific urban climate and a related weather file can be generated. These files allow to more accurate performance simulations of buildings placed in the urban environment. The importance of the PCA analysis in avoiding correlations between different urban variables makes these data very useful for further studies in other locations.
